# 21例胸腺瘤相关纯红细胞再生障碍患者的临床特征及预后

**DOI:** 10.3760/cma.j.issn.0253-2727.2023.12.011

**Published:** 2023-12

**Authors:** 田 张, 伊慧 赵, 丽娟 李, 化泉 王, 嘉 宋, 玉红 吴, 莉民 邢, 文 瞿, 国锦 王, 晶 关, 鸿 刘, 晓明 王, 宗鸿 邵, 蓉 付

**Affiliations:** 天津医科大学总医院血液科，天津 300052 Department of Hematology, Tianjin Medical University General Hospital, Tianjin 300052, China

纯红细胞再生障碍（pure red cell aplasia, PRCA）是指因骨髓中红系细胞显著减少或缺如所致的一种贫血性疾病，分为原发性和获得性[1]。胸腺瘤相关PRCA在获得性PRCA中所占比例最高[2]。胸腺瘤相关PRCA的发病可能源于胸腺自身免疫对造血的抑制[3]。本研究回顾性分析了我院胸腺瘤相关PRCA患者的临床特征及预后，以期为胸腺瘤相关PRCA的诊治提供更多的经验。

## 病例与方法

1. 病例资料：本研究回顾性分析了2005年1月1日至2022年11月1日我院收治的胸腺瘤相关PRCA患者共21例。所有病例均经组织病理学证实为胸腺瘤，根据骨髓穿刺等检查结果诊断为PRCA。PRCA的诊断参照《获得性纯红细胞再生障碍诊断与治疗中国专家共识（2020年版）》[4]，胸腺瘤的分型依据WHO对胸腺肿瘤组织学分型的分类[5]。

2. 治疗方式及疗效评价：本组中，19例患者行胸腺瘤切除术，2例患者单纯进行放疗。免疫抑制治疗方案包括：环孢素A（CsA）±激素、西罗莫司±激素以及单用激素治疗。采用《获得性纯红细胞再生障碍诊断与治疗中国专家共识（2020年版）》[4]进行疗效评价。治疗有效包括完全缓解（CR）和部分缓解（PR）。

3. 统计学处理：采用SPSS 26.0进行统计学分析。正态分布数据采用均数±标准差表示。偏态分布数据以中位数（范围）表示。使用Kaplan-Meier方法绘制生存曲线，事件为PRCA相关疾病进展或患者死于胸腺瘤，采用Log-rank检验进行组间比较。*P*<0.05为差异有统计学意义。

## 结果

一、胸腺瘤相关PRCA基本特征

21例患者中，男9例，女12例，中位年龄60（26～79）岁。1例（4.76％）伴重症肌无力。5例（23.81％）在胸腺瘤切除术后发生PRCA，距手术的中位时间为36（3～120）个月；1例（4.76％）在胸腺瘤复发时诊断PRCA，其余15例（71.43％）为发现胸腺瘤同时伴发PRCA。本组19例患者行胸腺瘤手术切除，根据WHO对胸腺肿瘤组织学分型的分类，AB型7例，A型2例，B型4例，8例组织学分型不详。CsA±激素组、西罗莫司±激素组和单用激素组治疗前HGB分别为（63.67±17.08）、（54.67±4.51）、（63.33±22.50）g/L，胸腺瘤手术切除组和非手术切除组治疗前HGB分别为（61.53±16.62）、（70.00±15.56）g/L。

二、胸腺瘤相关PRCA疗效分析

1. 胸腺瘤的外科治疗：本研究中，19例（90.48％）患者进行了胸腺瘤切除术（其中2例手术联合放射治疗），2例（9.52％）患者单纯放射治疗。3例（15.78％）患者术后出现胸腺瘤复发，其中2例（66.67％）行第2次手术切除。本研究对9例患者术后血象进行监测评估，中位观察时间为27（8～48）d，所有患者均同时采取输血及促红细胞生成素（EPO）治疗。6例患者术后血象上升，其中2例HGB上升≥30 g/L；3例（33.33％）患者术后血象下降（表1）。

**表1:** 胸腺瘤相关纯红细胞再生障碍（PRCA）患者治疗方式及疗效

治疗方式及疗效	例数（%）
胸腺瘤治疗方式	
手术切除	19（90.48）
非手术切除（放疗）	2（9.52）
胸腺瘤结局	
复发或转移	4（19.05）
疾病缓解	17（80.95）
治疗方案	
环孢素A±激素	15（71.43）
西罗莫司±激素	3（14.29）
激素	3（14.29）
初始诱导治疗疗效	
完全缓解	13（61.90）
部分缓解	3（14.29）
未缓解	5（23.81）
复发情况	
复发	3（14.29）
无复发	18（85.71）
并发症	
肺部感染	8（38.10）
肾功能不全	3（14.29）
肝功能不全	1（4.76）
生存情况	
生存	17（80.95）
死亡	4（19.05）
死亡原因	
肺部感染	2（9.5）
胸腺瘤复发	1（4.8）
胸腺瘤转移	1（4.8）

2. 免疫抑制治疗疗效及影响因素：本研究的免疫抑制治疗包括CsA±激素、西罗莫司±激素及单用激素，总有效率（ORR）为76.19％（16/21）。CsA±激素组、西罗莫司±激素及单用激素组的ORR分别为86.67％、66.67％、33.33％；CR率分别为66.67％、66.67％、0。CsA组中位CR时间为60（11～195）d，西罗莫司±激素组2例患者分别在80 d、81 d达到CR。胸腺瘤手术切除组和非手术切除组的ORR分别为84.21％、0。免疫抑制治疗有效率在手术切除后胸腺瘤无复发、手术切除后胸腺瘤复发、非手术切除3组中呈逐步下降趋势（87.50％、66.67％、0）。CD4/CD8≤1、高铁蛋白水平（血清铁蛋白>2 000 µg/L）患者免疫抑制治疗有效率降低（50.00％对100.00％，0对88.89％）。

有4例患者合并淋巴细胞增殖性疾病（19.05％）。2例患者合并T大颗粒淋巴细胞白血病（T-LGLL），予西罗莫司治疗获得持续缓解。1例患者合并T淋巴细胞增殖性疾病，先后接受激素、西罗莫司、西达本胺+激素治疗均无效。1例患者合并NK大颗粒淋巴细胞白血病（NK-LGLL），先后应用CsA、西罗莫司、甲氨蝶呤治疗无效。

3. 胸腺瘤相关PRCA复发情况：在13例CsA首次诱导治疗有效的患者中，3例（23.08％）出现PRCA复发，均因停用CsA，再次加用免疫抑制剂均有效；4例（30.77％）患者停用CsA后保持持续缓解，CsA中位维持时间为42.5（27～45）个月。

4. 并发症：在本研究中，最常见的并发症是肺部感染（8例，38.10％），其中2例患者死亡。3例（14.29％）出现肝功能不全，1例（4.76％）出现肾功能不全。

三、生存分析

中位随访50（15～176）个月，共有4例（19.05％）患者死亡。13例PRCA治疗有效患者OS率为100.0％，7例PRCA治疗无效组OS率为（21.4±18.8）％，差异有统计学意义（*χ*^2^＝12.213，*P*<0.001）；17例胸腺瘤无复发或转移患者OS率为（94.1±5.7）％，4例胸腺瘤复发或转移患者OS率为（25.0±21.7）％，差异有统计学意义（*χ*^2^＝6.868，*P*<0.009）。提示PRCA疗效及胸腺瘤复发或转移对胸腺瘤相关PRCA患者的生存可能存在影响。

## 讨论

胸腺瘤相关PRCA在继发性PRCA中占7％～10％[6]。首选治疗为胸腺瘤切除术联合免疫抑制治疗，有效率60％～80％[7]。胸腺瘤相关PRCA通常被认为是一种器官特异性自身免疫性疾病，研究发现，在胸腺瘤中T细胞的阳性选择和阴性选择缺陷，可导致自身免疫性疾病的发生[8]。PRCA的发生可能与胸腺瘤产生抑制性T细胞抑制红系分化有关[9]，胸腺和成红细胞可能共用同一种抗原，在胸腺中形成了抗成红细胞或促红细胞生成素的自身抗体，从而抑制造血[3]；胸腺瘤也可能通过T细胞介导的对红系祖细胞或前体细胞的损伤而引起骨髓抑制[10]。然而，胸腺瘤相关PRCA的确切机制仍待进一步探究[3],[9]。

大多数胸腺瘤相关PRCA患者在1个月内发现PRCA和胸腺瘤，也有部分患者PRCA发生在胸腺瘤切除后，尤其是在胸腺瘤复发的情况下[11]。对于胸腺瘤切除后的患者，特别是晚期或有复发风险者，随访中应注意发生PRCA的可能。继发PRCA的胸腺瘤组织学类型以AB型最常见[12]。胸腺瘤相关PRCA患者可同时伴有其他自身免疫性疾病。

先前的研究表明胸腺瘤外科切除可以使25％～30％胸腺瘤相关PRCA患者达到缓解，为首选治疗方式，对于胸腺瘤Ⅳ期或手术不能完全切除的患者，术后应辅以放疗[11]。然而，通常手术切除联合或不联合放疗不能使患者血象恢复正常，术后仍需要进行免疫抑制治疗[13]。大多数胸腺瘤术后复发是局部和区域性的，胸腺瘤组织学分型、病理阶段和初次手术切除不完全是胸腺瘤复发的常见因素[14]。我们的结果提示，胸腺瘤手术切除后需要联合应用免疫抑制治疗，胸腺瘤的去除与否以及胸腺瘤是否复发，都可能影响胸腺瘤相关PRCA的疗效。

胸腺瘤相关PRCA的治疗还包括免疫抑制治疗和对症支持治疗。既往研究显示，CsA在95％的胸腺瘤相关PRCA患者中有效[15]。近年来，西罗莫司治疗获得性PRCA多有报道，可使部分难治或CsA不耐受者获益。其可能的机制为西罗莫司抑制抗原诱导的T细胞增殖，并上调PRCA患者的调节性T细胞（Treg）水平，从而恢复造血[16]–[17]。本研究结果显示，含有CsA的方案ORR为86.67％，含有西罗莫司的方案ORR为66.67％。

此外，胸腺瘤相关PRCA患者外周血可观察到B淋巴细胞计数下降、CD4^+^ T淋巴细胞数量降低、CD8^+^ T淋巴细胞数量升高，CD4^+^/CD8^+^比值倒置[18]。铁过载可增加活性氧的产生，活性氧对造血有显著抑制作用[19]–[20]。本研究结果与上述文献一致，提示对于胸腺瘤相关PRCA患者，应监测CD4/CD8比值及铁蛋白水平。

中断CsA治疗与PRCA复发密切相关[21]。研究发现，CsA治疗达到缓解后，缓慢减量可以减少PRCA的复发[22]。本研究显示，PRCA复发与CsA初始治疗不规律及自行减量可能相关。同时，本研究提示，即使行胸腺瘤切除术，部分患者在停用CsA维持治疗后，仍出现复发。长期接受免疫抑制剂治疗应警惕感染风险[23]，预防和适当处理感染并发症在胸腺瘤相关PRCA治疗过程中非常重要，必要时应定期监测患者免疫功能。

胸腺瘤相关PRCA患者发生严重和反复感染时应考虑到Good综合征（GS）可能[23]。GS是胸腺瘤引起的副肿瘤性自身免疫并发症，其突出特征是低丙种球蛋白血症、B细胞减少或缺失以及T细胞减少。定期补充IgG是其理想的治疗方式，可以积极控制感染并降低死亡率[24]。对于胸腺瘤相关PRCA患者应注意监测血清免疫球蛋白水平和外周血B细胞数量，提高对GS的早期诊断及干预，从而改善患者预后。

PRCA与淋巴细胞增殖性疾病的相关性值得关注。克隆性T细胞不仅在T-LGLL相关的PRCA中发现，也存在于胸腺瘤相关PRCA中[25]。胸腺瘤相关PRCA患者可能存在与T-LGLL患者相似的克隆性T细胞增殖[26]。对于胸腺瘤相关PRCA患者，不论胸腺瘤术后有无复发，应警惕合并淋巴细胞增殖性疾病的可能，特别是对免疫抑制治疗无效患者，建议进行流式及相关基因检查。

胸腺瘤伴发PRCA后，预后较单纯胸腺瘤患者差，其常见的死亡原因是胸腺瘤进展、肺炎等[6]。研究表明，诱导免疫抑制治疗无效、复发是胸腺瘤相关PRCA患者死亡的危险因素[7]。本研究亦观察到免疫抑制治疗的疗效不佳、胸腺瘤复发转移可能影响胸腺瘤相关PRCA的预后。
